# YBYRÁ facilitates comparison of large phylogenetic trees

**DOI:** 10.1186/s12859-015-0642-9

**Published:** 2015-07-01

**Authors:** Denis Jacob Machado

**Affiliations:** Inter-institutional Grad Program on Bioinformatics, University of São Paulo, Rua do Matão, tv. 14, no. 101, sala 137, São Paulo, 05508-090 Brazil

**Keywords:** Diagnostic character states, Rogue taxa, Sensitivity analysis, Tree comparison

## Abstract

**Background:**

The number and size of tree topologies that are being compared by phylogenetic systematists is increasing due to technological advancements in high-throughput DNA sequencing. However, we still lack tools to facilitate comparison among phylogenetic trees with a large number of terminals.

**Results:**

The “YBYRÁ” project integrates software solutions for data analysis in phylogenetics. It comprises tools for (1) topological distance calculation based on the number of shared splits or clades, (2) sensitivity analysis and automatic generation of sensitivity plots and (3) clade diagnoses based on different categories of synapomorphies. YBYRÁ also provides (4) an original framework to facilitate the search for potential rogue taxa based on how much they affect average matching split distances (using MSdist).

**Conclusions:**

YBYRÁ facilitates comparison of large phylogenetic trees and outperforms competing software in terms of usability and time efficiency, specially for large data sets. The programs that comprises this toolkit are written in Python, hence they do not require installation and have minimum dependencies. The entire project is available under an open-source licence at http://www.ib.usp.br/grant/anfibios/researchSoftware.html.

## Background

Phylogenetic trees comprising hundreds or thousands of terminals are becoming increasingly common [1], and technological breakthroughs in high throughput DNA sequencing promise to allow trees to expand even more [2]. Within this context, there is an increasing demand for software solutions that help phylogeneticists to automate the process of comparing multiple optimal or nearly optimal topologies as well as topologies derived from different data partitions, optimality criteria, or assumption sets and extract information about the distribution of evidence in those trees [3]. The “YBYRÁ” package was developed to allow researchers to compare multiple trees containing large numbers of terminals quickly and accurately.

## Implementation

YBYRÁ is written in Python; hence, it is a cross-platform application (e.g., Windows, OS X or Linux) and does not require compilation. YBYRÁ makes use of free, easy to install Python modules to root trees and print images in SVG format. Search for potential wildcard taxa and the identification of diagnostic character states requires MSdist v0.5 [4] and TNT v1.1 [5], respectively. The programs, examples files and a graphic user interface for creating and editing configuration files can be downloaded under the GNU General Public License version 3.0 (GPL-3.0) at http://www.ib.usp.br/grant/anfibios/researchSoftware.html. A wiki page is available at https://gitlab.com/MachadoDJ/ybyra/wikis/home.

### Topological distances

Topological distance algorithms implemented in YBYRÁ are based on [6] and are highly sensitive to displacement of an insignificant number of terminals (see discussion in [4]). Althought there are already many programs (e.g., APE [7]) in which topologocal distance calculation is implemented, I believe the user may find it convenient to have this implemented in the same package as the functions described bellow. By *T* I denote the set of binary trees *T*={*T*_1_,*T*_2_...*T*_*n*_} given in the configuration file. Each of those trees is composed of a set of limited elements *E*_*n*_ (splits or clades, chosen by the user). The local distance *d* between *T*_1_ and *T*_2_ is defined by Equation 1. The global distance *D* between *T*_1_ and *T*_2_ is defined by Equation 2. Distance values will vary from zero to one. The lower the number of shared clades or splits, the greater the distance values.

(1) $$\begin{array}{*{20}l} d_{\left(T_{1},T_{2}\right)}&=1-\left(\frac{E_{1} \cap E_{2}}{E_{1} \cup E_{2}}\right) \end{array} $$

(2) $$\begin{array}{*{20}l} D_{\left(T_{1},T_{2}\right)}&=1-\left(\frac{E_{1} \cap E_{2}}{\bigcup_{i\in n} E_{i}}\right) \end{array} $$

**Fig. 1:**
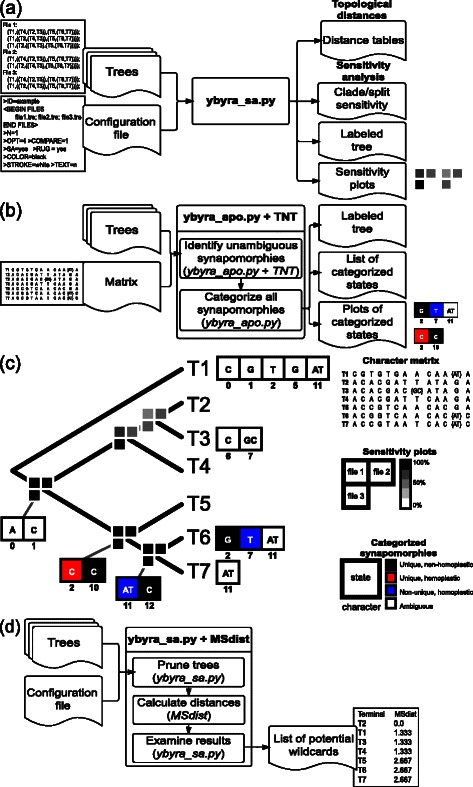
An example implementation of YBYRÁ. **a** Distance calculation and sensitivity analysis. **b** Categorization of synapomorphies. **c** SVG plots are named according to node numbers to facilitate manual image editing. **d** Search for wildcard taxa

The input for topology comparison and distance calculation consists of a configuration file and one or more files with trees in Newick format. A simplified flowchart for the entire operation is depicted in Fig. 1a. The process to calculate topological distances and perform sensitivity analysis is similar (see bellow) and both can be executed simultaneously. Tree distance calculation using all the clades in 100 trees with 1000 terminals each takes approximately 3.5 minutes and requires less than 80 MB of memory using a common personal computer (2.9 GHz Intel Core i7 with memory of 8 GB, 1600 MHz DDR3).

### Sensitivity analysis

In phylogenetic systematics, authors make use sensitivity analysis to address how much hypothesis choice may be affected by variables such as different tree search strategies, optimality criteria, alignment methods, and transformation cost schemes [8–10]. There is some debate in the literature regarding the scientific and heuristic value of sensitivity analysis [11, 12]. However, the instrumental value of sensitivity analysis as means to describe and compare different methodological approaches in systematics is indisputable.

Sensitivity analysis can be performed to evaluate how results depend on assumptions such as analytical parameters, search strategies, optimality criteria, alignment methods, and transformation costs. The input for sensitivity analysis is the same as above and a simplified flowchart for the entire process is shown in Fig. 1a). Although there is already a program dedicated to sensitivity analysis (i.e., “Cladescan” [13]), the user may find YBYRÁ useful due to its velocity and use of resources. I compared speed and memory usage of YBYRÁ versus Cladescan using three different data sets with 10, 100 and 1,000 terminals for 1,000, 100 and 10 trees respectively (Table 1). All timings were performed using a personal computer (see configuration above). Although both programs have the same results, YBYRÁ outperforms Cladescan in terms of CPU seconds and wall time. YBYRÁ will use significantly less memory than Cladescan when trees have a large number of terminals.

**Table 1:** Comparing the approximate execution time and memory use of Cladescan and YBYRÁ (2.9 GHz Intel Core i7, 8 GB 1600 MHz DDR3)

No. of	No. of	CPU	Wall	Memory
terminals	trees	time	time	use
Cladescan				
10	1000	3.532 sec	3.537 sec	3.4 MB
100	100	330.583 sec	331.151 sec	7 MB
1000	10	>9 h	>9 h	>2.7 GB
YBYRÁ				
10	1000	1.2291 sec	1.376 sec	8.7 MB
100	100	2.4297 sec	2.862 sec	14.7 MB
1000	10	78.6577 sec	81.857 sec	106.8 MB

### Evaluation of diagnostic character states

Differing from character-based DNA barcoding approaches such as CAOS [14], YBYRÁ categorizes character transformation events from any source of data given all possible optimization schemes in a set of trees. The input consists of one or more trees in TREAD format and a matrix in simplified NEXUS format containing a single DATA block. YBYRÁ proceeds by spawning tree(s) and data matrix to TNT to compile synapomorphies using TNT’s command “apo”. Synapomorphies are categorized as ambiguously or unambiguously optimized. Unambiguously optimized synapomorphies are further classified as unique and non-homoplastic, unique and homoplastic or non-unique and homoplastic. Program output consists of a table in comma-separated-values (CSV) and vector graphic files (SVG) illustrating categorized character states (Fig. 1b; see manually edited tree in Fig. 1c).

### Detection of wildcard taxa

In phylogenetic analysis, lack of data or conflicting information may cause some terminals to be highly unstable “wildcards” or “rogues” (see [3] for a recent empirical example). YBYRÁ offers a framework to rank every terminal according to how much it affects the average matching split distances (MSD) calculated in MSdist. Trees are pruned one terminal at a time and submitted to MSdist. YBYRÁ will generate an ordered list of terminals according to how much they affect MSD (see Fig. 1d). Terminals that resulted in the lowest MSD are more likely to cause decrease of resolution and may be considered potential wildcard.

In [3], the author’s used homemade scripts to prune terminals from the set of most parsimonious trees, recalculate the strict consensus using TNT and count the number of nodes nodes in a iterative manner. YBYRÁ automates this process and was able to recover the same results with fewer commands.

## Discussion

YBYRÁ is dedicated to phylogeneticists with minimal computational skills. To facilitate usage, it accompanies a graphic user interface to create and edit configuration files and the user receives instructions in case additional modules are required to run specific functions. The package integrates strategies for topological comparison and distance calculation, as well as a novel framework to search for potential rogue taxa. It also offers a different strategy to compile and evaluate diagnostic character states than CAOS. While CAOS aims to identify diagnostic character states from molecular sequences without reference to tree topology, YBYRÁ uses TNT to categorize all transformation events considering every possible optimization scheme in the observed trees. Finally, YBYRÁ outperforms Cladescan for phylogenetic sensitivity analysis, allowing automatic generation of sensitivity plots for large data sets in feasible time.

## Conclusion

The present project provides user-friendly programs that allows automatization and reproducibility of result analysis operations in phylogenetics. To of my knowledge, YBYRÁ is the first software package to integrate solutions for topological distance calculation, extraction of diagnostic characters and search for potential rogue taxa. Additionally, it outperforms Cladescan for the analysis of large data sets and is currently the only viable solution for automated phylogenetic sensitivity analysis of large trees (over 1.000 terminals).

## Availability and requirements

**Project name:** YBYRÁ**Project home page:** http://www.ib.usp.br/grant/anfibios/researchSoftware.html **Operating system(s):** Windows, Linux, OS X**Programming language:** Python**Licence:** GNU General Public License version 3.0(GPL-3.0)**Other requirements:** view dependencies in thedocumentation.**Any restrictions to use by non-academics:** view license.
